# Disparities in Advance Care Planning: Did COVID‐19 Change Anything?

**DOI:** 10.1111/jgs.70509

**Published:** 2026-05-13

**Authors:** Anupama Gangavati, Kimberly S. Johnson, Alyssa Platt, Maren Olsen, Raegan W. Durant, Deborah Ejem, Marie Bakitas, Rowena Dolor, Sherone N. Williams‐Bryant, Nadine Barrett, Ronit Elk, Tammie Quest, Marisette Hasan, Kenisha Bethea, Ramona Rhodes

**Affiliations:** ^1^ Department of Internal Medicine, Division of Geriatric Medicine University of Texas at Southwestern Medical Center Dallas Texas USA; ^2^ Division of Geriatrics, Department of Medicine Duke University, Geriatric Research Education and Clinical Center, Durham Veterans Affairs Health Care System Durham North Carolina USA; ^3^ Department of Biostatistics and Bioinformatics Duke University Durham North Carolina USA; ^4^ Division of General Internal Medicine and Population Science Heersink School of Medicine, University of Alabama at Birmingham Birmingham Alabama USA; ^5^ School of Nursing, University of Alabama at Birmingham Birmingham Alabama USA; ^6^ School of Nursing, Heersink School of Medicine Center for Palliative and Supportive Care, University of Alabama at Birmingham Birmingham Alabama USA; ^7^ Division of General Internal Medicine, Department of Medicine Duke University School of Medicine Durham North Carolina USA; ^8^ Atrium Health/Wake Forest Comprehensive Cancer Center, Wake Forest School of Medicine Winston‐Salem North Carolina USA; ^9^ Heersink School of Medicine Division of Geriatrics, Gerontology, and Palliative Care, Center for Palliative and Supportive Care, University of Alabama at Birmingham Birmingham Alabama USA; ^10^ Department of Emergency Medicine Emory University Atlanta Georgia USA; ^11^ Department of Preventive Medicine Emory University Atlanta Georgia USA; ^12^ South Carolina Coalition for the Care of the Seriously Ill Columbia South Carolina USA; ^13^ Clinical and Translational Science Institute, Duke University Durham North Carolina USA; ^14^ University of Texas at Southwestern Medical Center Department of Internal Medicine, Division of Geriatric Medicine, O'Donnell School of Public Health Dallas Texas USA

**Keywords:** advance care planning, COVID‐19, disparities, end‐of‐life care preferences

## Abstract

**Background:**

Rates of advance care planning (ACP) are lower and preferences for life‐prolonging treatment are higher among Black compared to White older adults. We examined whether these differences persisted during the COVID‐19 pandemic.

**Methods:**

Between February 2021 and September 2022, we conducted a cross‐sectional COVID‐19–focused survey of seriously ill adults ≥ 65 years in 10 primary care clinics participating in a clinical trial of two ACP interventions. Logistic regression models examined associations between COVID‐19 related ACP discussion (defined as discussions with family, friends, or doctors about COVID‐related medical care) and treatment preferences if very sick with COVID‐19 (life‐prolonging treatment, comfort care, trial of life‐prolonging with transition to comfort care if no improvement) overall and by race, controlling for baseline characteristics.

**Results:**

Among 428 participants (55.9% Black, 44.2% White; mean age 74.6), 25% reported discussing COVID‐19 treatment preferences with family/friends and 6% with doctors. Most reported no change in willingness to participate in ACP due to the pandemic, though increased willingness was more common among Black than White participants (22.4% vs. 14.0%, *p* = 0.016). Despite this, COVID‐19‐related ACP discussion did not differ by race (family/friends: 22.7% vs. 28.3%, *p* = 0.19; doctors: 6.9% vs. 4.8%, *p* = 0.37). Most seriously ill older adults preferred a time‐limited trial of life‐prolonging treatment (71% White, 56.2% Black); though preferences varied by race (*p* < 0.0001); Black participants compared to White participants more often preferred life‐prolonging treatment (28.5% vs. 10.3%). In adjusted models, race was not associated with COVID‐19‐related ACP discussion (OR 0.72, 95% CI 0.44–1.18), while preferences for life‐prolonging treatments predicted greater COVID‐19 related ACP discussion (OR 1.98, 95% CI 1.14–3.44).

**Conclusion:**

In contrast to pre‐pandemic ACP research, no racial differences were observed in COVID‐19‐related ACP discussion, though differences in treatment preferences persisted. These findings underscore the need for culturally responsive, context‐sensitive ACP approaches among seriously ill older adults.

## Summary

- Key points
  ○ In this study of 428 seriously ill Black and White older adults, only 25% talked to family or friends, and 6% talked to healthcare providers about the type of care they would want if they became sick with COVID‐19. Rates of COVID‐19‐related advance care planning discussions were similar among seriously ill Black and White older adults.
  ○ Among these seriously ill older adults, most preferred a trial of life‐prolonging treatments with a transition to comfort care if no improvement if they became very sick with COVID‐19 (71% of White older adults and 56% of Black older adults). Preferences for all treatments to stay alive as long as possible if very sick with COVID‐19 were expressed by a small proportion of seriously ill older adults and were more common among Black older adults (28.5% vs. 10.3%).
  ○ Seriously ill older adults who wanted all treatments to stay alive as long as possible (vs. comfort‐focused care or trial of life‐prolonging therapies with a change to comfort care if no improvement) were more likely to have talked to family, friends, or doctors about their treatment preferences if they became very sick with COVID‐19.
- Why this paper matters?
  ○ Most studies have found lower rates of advance care planning (ACP) among Black compared to White seriously ill older adults. In this study, however, rates of COVID‐related ACP discussions were similar among seriously ill Black and White older adults. Preferences for care if very sick with COVID‐19, however, varied by race. Although most older adults preferred a time‐limited trial of life‐prolonging treatment, Black older adults were more likely than White older adults to prefer all life‐prolonging treatments. These findings suggest that even when rates of ACP‐related discussions are similar, racial differences in preferences for care persist among seriously ill older adults. These findings highlight the need for interventions that both promote ACP discussion outside of crisis contexts and honor the cultural values that shape end‐of‐life care preferences.

## 1 Introduction

Advance care planning (ACP) helps adults understand and share their values and preferences for future medical care [1, 2, 3]. ACP improves end‐of‐life communication, documentation of care preferences, and reduces surrogate distress [4, 5, 6]. Despite these benefits, only about half of older adults engage in ACP with only one‐third documenting their wishes [1, 5, 7, 8].

The COVID‐19 pandemic intensified the need for ACP, especially for older adults with serious illness facing prognostic uncertainty, high mortality risk, and potential constraints on life‐prolonging therapies due to workforce and resource shortages [9, 10, 11]. In response, health systems implemented ACP strategies, using telehealth, online tools, and public awareness, increasing some ACP engagement [12, 13].

However, the benefits of ACP and the burdens of COVID‐19 were unevenly distributed [5]. Black Americans faced disproportionately high COVID‐19 morbidity and mortality early in the pandemic [14] and have historically lower rates of ACP discussions than White Americans [10, 12]. It is unclear how pre‐existing ACP disparities shaped pandemic‐era ACP discussions. Black patients are also more likely to want life‐sustaining therapies even when prognosis is poor, but how COVID‐19 influenced these preferences is poorly understood [7, 8, 9, 10, 13].

To address these gaps, we analyzed data from the Reducing Disparities in the Quality of Palliative Care for Older African Americans through Improved ACP (EQUAL ACP) study, a multi‐site, cluster randomized trial comparing the effectiveness of two ACP interventions among Black and White older adults with serious illness [10]. The COVID‐19 survey was administered between February 2021 and September 2022 during the parent trial to assess pandemic‐specific experiences, including ACP discussion defined as discussions with family, friends, or doctors about preferences for COVID‐19‐related medical care. We hypothesized that pre‐existing racial differences in ACP discussion and treatment preferences would persist during the pandemic, with minimal pandemic‐related change.

## 2 Methods

This cross‐sectional analysis includes baseline and COVID‐related survey data from the EQUAL ACP study, which enrolled older adults with serious illness from 10 outpatient clinics across five southern states (Duke in Durham, NC, University of Alabama at Birmingham, Emory in Atlanta Georgia, UT Southwestern in Dallas Texas, and two Federally Qualified Health Centers (FQHC) in Aiken and Beaufort, South Carolina) and was conducted from January 2019 through March 2024. Data were collected at enrollment and at 3, 6, and 12 months. A COVID‐related survey was added for new or existing participants completing a survey between February 2021 and September 2022. Most COVID‐19 surveys occurred at enrollment (baseline, prior to ACP intervention) (64.7%), with a similar distribution by race (*p* = 0.77). (Table S1). The study protocol was previously published [15] and approved by institutional review boards of participating institutions. All participants gave informed consent and received $50 per survey.

### 2.1 Participants

Eligible participants were ≥ 65, Black or White race, cognitively able to participate, community‐dwelling, had ≥ 2 visits to a participating clinic in the past year, no EHR‐documented ACP, no hospice or outpatient palliative care use, and a serious illness diagnosis. Eligible conditions included metastatic cancer or hematologic malignancy; chronic obstruction pulmonary disease (COPD), interstitial lung disease, or Stage III/IV Congestive heart failure and on home oxygen or hospitalization in the last year; chronic kidney disease Stage 5 or on dialysis; advanced liver disease or cirrhosis; diabetes with complications (heart disease, peripheral vascular disease, renal disease); two or more unplanned hospitalizations in the last year; > age 70 with hip fracture in the last year. In addition, patients > age 75 were eligible if they were dependent in one or more activities of daily living; had > 2 comorbidities above that did not meet criteria for advanced disease (ex: non‐metastatic cancer on treatment or COPD but not requiring oxygen or recently hospitalized); or if they were referred by their primary care provider who answered no to the “surprise question” (Would you be surprised if this patient died in the next year?).

### 2.2 Measures—COVID‐19 Survey

Participants completed a COVID‐19 survey which assessed: (1) change in willingness (more, less, no change) due to the pandemic to discuss care preferences if unable to speak for themselves; (2) whether they had discussed COVID‐19 related treatment preferences with family, friends, or doctor (main outcome); (3) preferences for care if sick with COVID‐19 (main exposure)—all treatments to live as long as possible versus comfort‐focused care and trial of life‐prolonging treatments with transition to comfort care if no improvement (Text S1).

We included participant age, sex, race, marital status, education, diagnoses, financial status and self‐rated health from the baseline survey.

### 2.3 Statistical Analysis

We hypothesized consistent with pre‐pandemic literature: (1) Black seriously ill older adults would be less likely than White seriously ill older adults to participate in COVID‐19–related ACP discussions and more likely to prefer life‐prolonging treatment if sick with COVID‐19 [7, 8, 16]. (2) Preference for life‐prolonging treatment would be associated with lower likelihood of COVID‐19 related ACP discussions, particularly among Black participants [2, 6, 17].

Sociodemographic and clinical characteristics were summarized using means and standard deviations for continuous variables, and frequency counts and percentages for categorical variables. Chi‐Square tests (or Fischer's Exact tests for expected cell counts < 5) and Wilcoxon rank sum tests were used to test differences in characteristics by race for categorical and continuous variables, respectively. Multivariable logistic regression was used to explore whether preferences for care if respondents were very sick with COVID‐19 (all medical treatments to keep you alive as long as possible vs. other choices) were associated with COVID‐19‐related ACP discussion (talking to family, friends, or doctors about COVID‐19 related preferences for care). A second model examined whether this association varied by race (race × treatment preference interaction). Based on prior research [7, 16, 18, 19], regressions were adjusted for age, sex, self‐rated health, marital status, education, and financial status. All analyses were conducted using SAS 9.4 (Cary, NC), and statistical significance was assessed at level *α* = 0.05.

## 3 Results

### 3.1 Baseline Characteristics

Of the 792 seriously ill participants completing baseline assessments in the EQUAL ACP study, 428 (55.9% Black, 44.2% White) completed a COVID survey during the study period (Figure S1). Mean age was 74.6 (6.3), and 64.7% were female. Among participants completing the COVID‐19 survey, Black participants were more likely to be female (72% vs. 55.6%), have diabetes with complications (56.5% vs. 37%), report difficulty paying bills (11.3% vs. 4.3%), and report fair/poor health (44.5% vs. 28.6%), and less likely to have a bachelor's degree or beyond (27.2% vs. 42.9%) (Table 1).

**TABLE 1:** Sample baseline characteristics.

	Black older adults (*N* = 239)	White older adults (*N* = 189)	Total (*N* = 428)	*p*
Age				0.020^a^
Mean (SD)	74.0 (6.3)	75.4 (6.3)	74.6 (6.3)	
Range	(65.0–91.0)	(65.0–94.0)	(65.0–94.0)	
Sex				< 0.001^b^
Male	67 (28.0)	84 (44.4)	151 (35.3)	
Female	172 (72.0)	105 (55.6)	277 (64.7)	
Diagnoses				
Metastatic or blood cancer	27 (11.3)	19 (10.1)	46 (10.7)	0.680^b^
Heart, lung, liver, and/or kidney disease	43 (18.0)	34 (18.0)	77 (19.0)	1.000^b^
Diabetes with complications	135 (56.5)	70 (37)	205 (47.9)	< 0.001^b^
Multimorbidity^c^	147 (61.5)	133 (70.4)	289 (65.4)	< 0.056^b^
Level of Education				0.001^b^
< High school	38 (15.9)	12 (6.3)	50 (11.7)	
Completed high school	47 (19.7)	33 (17.5)	80 (18.7)	
Vocational school/some College	89 (37.2)	63 (33.3)	152 (35.5)	
≥ Bachelor's degree	65 (27.2)	81 (42.9)	146 (34.1)	
Marital Status				< 0.001^b^
Married/Living with partner	76 (31.8)	102 (54.0)	178 (41.6)	
Other	163 (68.2)	87 (46.0)	250 (58.4)	
Household's financial situation right now?				< 0.001^b^
After bills, enough $ for special things	83 (34.9)	110 (58.5)	193 (45.3)	
Enough $ for bills, but little for special things	92 (38.7)	53 (28.2)	145 (34.0)	
Enough $ for bills, but only after cutting back	36 (15.1)	17 (9.0)	53 (12.4)	
Difficulty paying bills	27 (11.3)	8 (4.3)	35 (8.2)	
Self‐rated health				0.001^b^
Excellent/Very Good/Good	132 (55.5)	135 (71.4)	267 (62.5)	
Fair/Poor	106 (44.5)	54 (28.6)	160 (37.5)	

^a^ Wilcoxon rank sum test.

^b^ Chi‐Square test; for all other race comparisons.

^c^ Multimorbidity: ≥ age 75 with 2 more chronic conditions and/or ADL disability, or clinician referral based on expected prognosis of < 1 year; 2 missing responses for financial situation—one from each race; 1 missing self‐rated health (Black participant).

### 3.2 ACP Discussion

**FIGURE 1:**
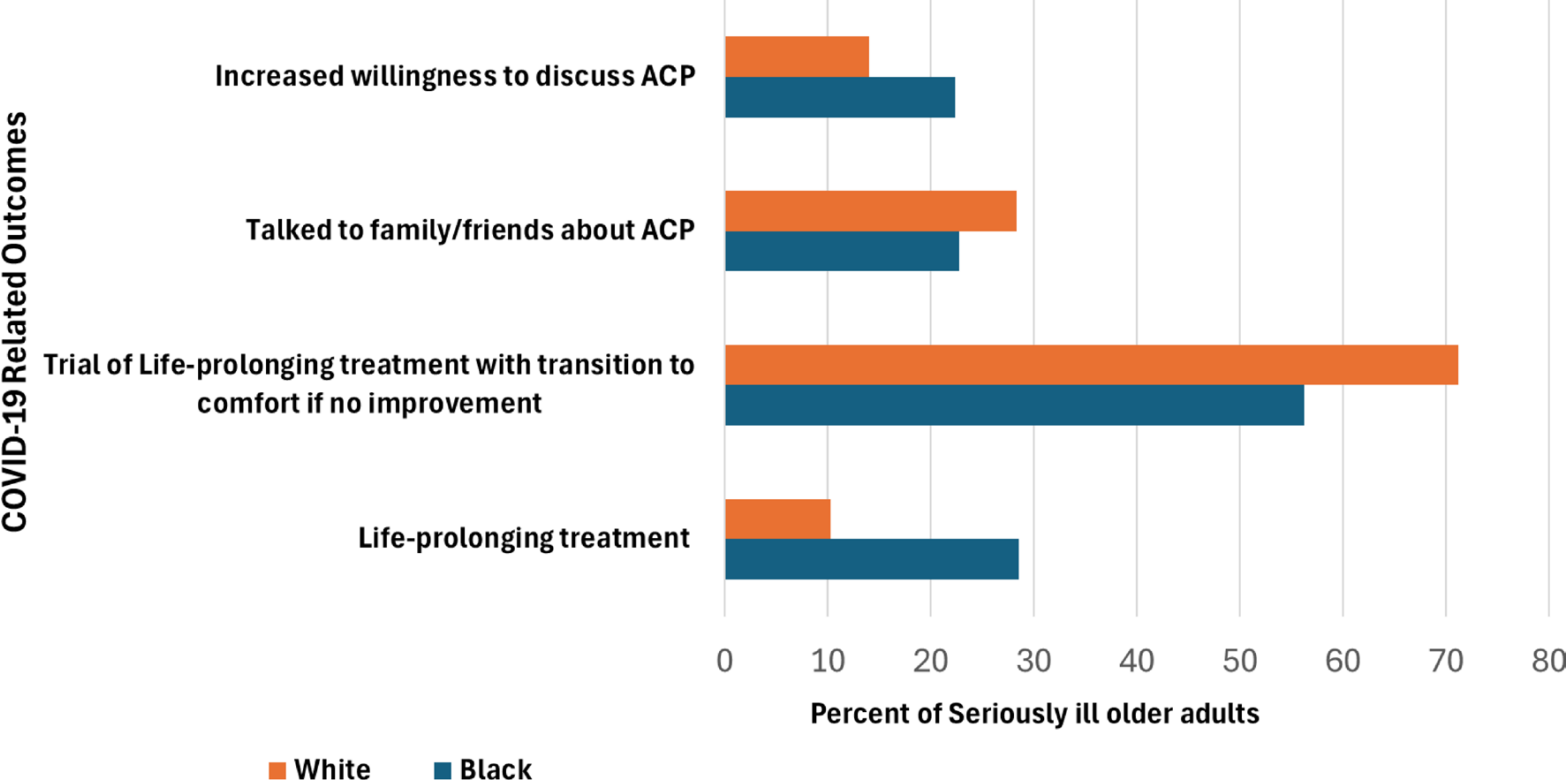
COVID‐19–related advance care planning and treatment preferences among seriously ill older adults. Bars show the percentage of Black and White seriously ill older adults who reported increased willingness to discuss advance care planning (ACP)^1^, participation in COVID‐19–related ACP discussions^2^ (defined as discussions with family, friends or doctors). Discussions with doctors were infrequent (6.9% among Black and 4.8% among White and therefore not shown separately). Bars also show preferences for care if very sick with COVID‐19^3^: Trial of life‐prolonging treatment with transition to comfort care if no improvement and life‐prolonging treatment. Comfort‐focused care preference was infrequent (8.3% Black; 10.9% White) and not shown separately. Percentages are shown by race. ^1^How has the COVID‐19 pandemic changed your willingness to talk to family, friends, or doctors about the medical care that you would want if you became too sick to speak for yourself? Compared to before the pandemic: Are you (a) more willing; (b) less willing; or (c) no change in your willingness to talk about your wishes? ^2^Have you talked to family or friends about the kind of medical care that you would want if you got COVID‐19? (a) Yes (b) No. Have you talked to your doctor about the kind of medical care that you would want if you got COVID‐19? Responses: (a) Yes (b) No ^3^If you became very sick with COVID‐19 and the doctors thought that you were not likely to recover, what kind of medical treatment would you want? Want all life prolonging measures (You would want all medical treatments to keep you alive as long as possible) versus all other responses: Want Comfort Measures (You would not want any medical treatments to prolong your life. Keep you comfortable and allow you to die naturally); Want life prolonging measures for a short time (You would want the doctors to try medical treatments to keep you alive, but if you do not improve, stop life support treatments and keep you comfortable; allow you to die naturally).

Most seriously ill older adults reported that the COVID‐19 pandemic did not change their willingness to discuss ACP with family, friends, or doctors. However, there was some variation by race (*p* = 0.016) with a greater proportion of Black older adults reporting that they were more willing to talk to family, friends, or doctors about their wishes (22.4% vs. 14.0%) due to the pandemic, (Figure 1), and a greater proportion of White participants reporting no change (86.0% vs. 76.3%). Twenty‐five percent reported talking to family or friends about the medical care that they would want if they had COVID, and only 6% reported that they had talked to a doctor; there were no significant differences by race. Being more willing to talk about wishes versus less willing or no change was associated with a greater likelihood of having talked to family, friends, or doctors about COVID‐related treatment preferences (35% vs. 21%, *p* = 0.0039); this relationship did not differ by race (*p* = 0.876) (Table S2).

### 3.3 Preferences for Care If Very Sick With COVID‐19

The majority (63.7%) of participants wanted a trial of life‐prolonging measures with a transition to comfort if no improvement; however, there was some variation by race (*p* < 0.001) (Figure 1). Black participants were more likely than White participants to want life prolonging measures to keep them alive as long as possible (28.5% vs. 10.3%) (*p* < 0.0001).

### 3.4 COVID‐Related ACP

In adjusted analysis, race (Black vs. White) was not associated with COVID‐related ACP discussions (Model 1, Table 2: AOR 0.72 95% CI 0.44, 1.18). Those who preferred all life‐prolonging measures (vs. other treatment preferences) were more likely to have participated in COVID‐related ACP discussions (AOR 1.98 95% CI 1.14, 3.44). The race‐by‐treatment preference interaction suggested that the association between wanting all life‐prolonging measures and participating in COVID‐related ACP was less pronounced in Black participants (AOR 0.25 95% CI 0.07, 0.84).

**TABLE 2:** Adjusted logistic regression of predictors of COVID‐19 related ACP discussion (talking to family, friends, or doctors about preferences for COVID‐related medical care) (*N* = 415)^a^.

Variable	Level	Model 1 odds ratio (95% CI) *p*	Model 2 odds ratio (95% CI) *p*
Intercept	—	1.61 (0.08, 30.78) *p* = 0.74	1.78 (0.09, 35.19) *p* = 0.71
Want all life‐prolonging measures^b^	No (Reference) Yes	1.98 (1.14, 3.44) *p* = 0.015	5.26 (1.90, 14.55) *p* = 0.0014
Race	White (Reference) Black	0.72 (0.44, 1.18) *p* = 0.189	0.94 (0.55, 1.62) 0.82
Race × want all life‐prolonging measures		—	0.25 (0.07, 0.84) *p* = 0.025

^a^ Adjusted for age, sex, self‐rated health, marital status, education, financial situation.

^b^ If you became very sick with COVID‐19 and the doctors thought that you were not likely to recover, what kind of medical treatment would you want? Want all life prolonging measures (You would want all medical treatments to keep you alive as long as possible) versus all other responses: Want Comfort Measures (You would not want any medical treatments to prolong your life. Keep you comfortable and allow you to die naturally); Want life prolonging measures for a short time (You would want the doctors to try medical treatments to keep you alive, but if you do not improve, stop life support treatments and keep you comfortable; allow you to die naturally).

Among White participants, 57.9% of those who wanted life prolonging therapies if very sick with COVID versus 24.2% of those with other preferences had talked to family, friends, or doctors (COVID‐related ACP) compared to 27.3% versus 22% of Black participants, respectively (Table S3).

## 4 Discussion

In this multi‐site cohort of community‐dwelling, seriously ill older adults, Black participants were more likely than White participants to report increased willingness to participate in ACP‐related discussions due to the pandemic. However, most older adults reported no change, and actual ACP‐related discussions remained low across both groups. If very sick with COVID‐19, most preferred a time‐limited trial of life‐prolonging treatment with transition to comfort care. Only one‐fifth of participants preferred all life‐prolonging treatments, and this was more common among Black participants. Preferences for life‐prolonging treatment were associated with greater ACP‐related discussions in both groups, though this association was weaker among Black participants. These findings highlight the role of context in shaping ACP‐related discussions and the relative stability of treatment preferences.

Despite efforts to expand ACP during the pandemic, ACP‐related discussions in our sample remained low. Only 25% of participants discussed COVID‐related treatment preferences with family or friends and 6% with doctors, consistent with prior studies of hospitalized COVID‐19 patients showing low rates of documented ACP conversations. This suggests persistent barriers to ACP‐related discussions even amid heightened risk and system‐level ACP initiatives [10, 13].

Although the pandemic may have heightened ACP awareness among Black patients due to the widely publicized higher COVID‐19 risk, a smaller, nonsignificant proportion of Black than White patients reported ACP‐related discussions (22.7% vs. 28.3%). This mismatch may reflect lower baseline rates of ACP discussions among Black patients and ongoing barriers, including less awareness about ACP and concerns about how ACP may affect care [7, 8].

In our sample, a majority of both Black and White older adults preferred a time‐limited trial of life‐prolonging treatment if sick with COVID, underscoring the need to avoid assumptions about treatment preferences based on race. While preferences for life‐prolonging treatments to live as long as possible were expressed by only one‐fifth of older adults, consistent with prior research [2, 9, 13, 20], these preferences were more common among Black participants (28.5% vs. 10.3%), highlighting the persistence of some racial differences even during a global health crisis [16]. This may reflect entrenched cultural, spiritual [13, 21, 22], and historical influences, including longstanding inequities in healthcare access and trust in the healthcare system due to past injustices and ongoing disparities.

Unexpectedly, in our cohort, participants preferring life‐prolonging treatment were more likely to engage in ACP‐related discussions. Typically, ACP is associated with limiting unwanted interventions [10, 20], but during the pandemic, concern about access to intensive treatments—like ventilators [11] may have motivated discussions to ensure care. This effect appeared stronger among White participants, possibly reflecting shifts from pre‐existing comfort‐focused preferences [5], and new concerns about the influence of ageism and resource rationing on COVID‐related care [11]—both of which may have increased the desire to share care preferences. Among Black participants, long‐standing concerns around care quality and stable preferences may explain the weaker association between ACP‐related discussions and life‐prolonging treatment [23, 24].

There are several limitations. The survey was administered from Feb 2021–Sept 2022, when treatments and vaccines were available, potentially influencing ACP‐related discussions. Participants were part of a larger trial to reduce ACP disparities, potentially influencing responses. As a cross‐sectional study, we could not assess changes over time, and findings may not generalize to populations without primary care access. Treatment preferences were assessed using a hypothetical scenario within a survey rather than through a facilitated ACP discussion. Thus, preferences may not reflect fully informed ACP decision‐making, though our findings align with existing literature. Prior COVID‐related ACP discussion was self‐reported, and our study did not assess ACP documentation in the electronic health record. Despite these limitations, inclusion of a majority‐Black sample (56.8%), a population historically underrepresented in end‐of‐life care research [25], adds to the study's importance. However, we cannot draw conclusions about other marginalized populations with lower ACP rates.

Our findings highlight the complex relationship among race, care preferences, ACP‐related discussions, and context, underscoring the need for person‐centered ACP that addresses systemic barriers and considers how factors, such as a global pandemic, may change patients' values and priorities. Future studies should evaluate context‐specific, culturally responsive interventions that foster equitable opportunities for ACP discussion and honor treatment preferences informed by “What matters most to patients.”

## Author Contributions

A.G.: study concept and design, acquisition, data interpretation, preparation of manuscript. K.S.J. and R.R.: study concept and design, data interpretation, preparation of manuscript. A.P. and M.O.: analysis (design and performance) and interpretation of data, preparation of manuscript. R.W.D., D.E., M.B., R.D., S.N.W.‐B., N.B., R.E., T.Q., M.H., K.B.: data interpretation and manuscript development.

## Funding

The Patient‐Centered Outcomes Research Institute (PCORI) funded this study, Contract Number R‐1609‐36381, Reducing Disparities in the Quality of Palliative Care for Older African Americans through Improved Advance Care Planning (EQUAL ACP Study). Dr. Kimberly S. Johnson served as the Principal Investigator. The statements presented in this publication are solely the responsibility of the authors and do not necessarily represent the views of the Patient‐Centered Outcomes Research Institute (PCORI), its Board of Governors, or Methodology Committee.

## Disclosure

The sponsor had no role in the design, methods, subject recruitment, data collection, analysis, or preparation of the manuscript. The Patient‐Centered Outcomes Research Institute (PCORI) funded this study, Contract Number R‐1609‐36381, Reducing Disparities in the Quality of Palliative Care for Older African Americans through Improved Advance Care Planning (EQUAL ACP Study). Dr. Kimberly S. Johnson served as the Principal Investigator. The statements presented in this publication are solely the responsibility of the authors and do not necessarily represent the views of the Patient‐Centered Outcomes Research Institute (PCORI), its Board of Governors, or Methodology Committee.

## Conflicts of Interest

The authors declare no conflicts of interest.

## Supporting information


**Figure S1:** Participant flow diagram.
**Table S1:** Patient characteristics by race.
**Table S2:** Frequency of talking to friends, family, or doctor about COVID‐19 related treatment preferences by willingness to talk, by race (*n* = 416).
**Table S3:** Cross‐tabulation of preference for life‐prolonging treatment by discussions with friends, family, or doctors, stratified by race.
**Text S1:** EQUAL‐ACP brief COVID‐19 survey.

## References

[1] S. E. Hickman , H. D. Lum , A. M. Walling , A. Savoy , and R. L. Sudore , “The Care Planning Umbrella: The Evolution of Advance Care Planning,” Journal of the American Geriatrics Society 71, no. 7 (2023): 2350–2356, 10.1111/jgs.18287. 36840690 PMC10958534

[2] R. L. Sudore , D. K. Heyland , H. D. Lum , et al., “Outcomes That Define Successful Advance Care Planning: A Delphi Panel Consensus,” Journal of Pain and Symptom Management 55, no. 2 (2018): 245–255.e8, 10.1016/j.jpainsymman.2017.08.025. 28865870 PMC5794507

[3] J. A. C. Rietjens , R. L. Sudore , M. Connolly , et al., “Definition and Recommendations for Advance Care Planning: An International Consensus Supported by the European Association for Palliative Care,” Lancet Oncology 18, no. 9 (2017): e543–e551, 10.1016/S1470-2045(17)30582-X. 28884703

[4] C. Malhotra , M. Shafiq , and A. P. M. Batcagan‐Abueg , “What Is the Evidence for Efficacy of Advance Care Planning in Improving Patient Outcomes? A Systematic Review of Randomised Controlled Trials,” BMJ Open 19, no. 7 (2022): e060201, 10.1136/bmjopen-2021-060201.

[5] N. Fleuren , M. F. I. A. Depla , D. J. A. Janssen , M. Huisman , and C. M. P. M. Hertogh , “Underlying Goals of Advance Care Planning (ACP): A Qualitative Analysis of the Literature,” BMC Palliative Care 19, no. 1 (2020): 27. 32143601 10.1186/s12904-020-0535-1 PMC7059342

[6] L. J. Brighton and K. Bristowe , “Communication in Palliative Care: Talking About the End of Life, Before the End of Life,” Postgraduate Medical Journal 92, no. 1090 (2016): 466–470, 10.1136/postgradmedj-2015-133368. 27153866

[7] J. J. Sanders , M. T. Robinson , and S. D. Block , “Factors Impacting Advance Care Planning Among African Americans: Results of a Systematic Integrated Review,” Journal of Palliative Medicine 19, no. 2 (2016): 202–227, 10.1089/jpm.2015.0325. 26840857

[8] K. S. Johnson , “Racial and Ethnic Disparities in Palliative Care,” Journal of Palliative Medicine 16, no. 11 (2013): 1329–1334, 10.1089/jpm.2013.9468. 24073685 PMC3822363

[9] V. Bhatia , R. Geidner , K. Mirchandani , Y. Huang , and H. J. Warraich , “Systemwide Advance Care Planning During the COVID‐19 Pandemic: The Impact on Patient Outcomes and Cost,” NEJM Catalyst 2, no. 9 (2021): CAT.21.0188.

[10] B. L. Block , A. K. Smith , and R. L. Sudore , “During COVID‐19, Outpatient Advance Care Planning Is Imperative: We Need All Hands on Deck,” Journal of the American Geriatrics Society 68, no. 7 (2020): 1395–1397, 10.1111/jgs.16532. 32359075 PMC7267338

[11] T. W. Farrell , L. E. Ferrante , T. Brown , et al., “AGS Position Statement: Resource Allocation Strategies and Age‐Related Considerations in the COVID‐19 Era and Beyond,” Journal of the American Geriatrics Society 68, no. 6 (2020): 1136–1142. 32374440 10.1111/jgs.16537 PMC7267615

[12] E. G. Price‐Haywood , J. Burton , D. Fort , and L. Seoane , “Hospitalization and Mortality Among Black Patients and White Patients With Covid‐19,” New England Journal of Medicine 382, no. 26 (2020): 2534–2543, 10.1056/NEJMsa2011686. 32459916 PMC7269015

[13] A. E. Barnato , G. R. Johnson , J. D. Birkmeyer , J. S. Skinner , A. J. O'Malley , and N. J. O. Birkmeyer , “Advance Care Planning and Treatment Intensity Before Death Among Black, Hispanic, and White Patients Hospitalized With COVID‐19,” Journal of General Internal Medicine 37, no. 8 (2022): 1996–2002, 10.1007/s11606-022-07530-4. 35412179 PMC9002036

[14] Centers for Disease Control and Prevention , “COVID Data Tracker,” 2024, U.S. Department of Health and Human Services, CDC, https://covid.cdc.gov/covid‐data‐tracker.

[15] D. B. Ejem , N. Barrett , R. L. Rhodes , et al., “Reducing Disparities in the Quality of Palliative Care for Older African Americans Through Improved Advance Care Planning: Study Design and Protocol,” Journal of Palliative Medicine 22, no. S1 (2019): 90–100, 10.1089/jpm.2019.0146. 31486728

[16] M. Bazargan and S. Bazargan‐Hejazi , “Disparities in Palliative and Hospice Care and Completion of Advance Care Planning and Directives Among Non‐Hispanic Blacks: A Scoping Review of Recent Literature,” American Journal of Hospice & Palliative Care 38, no. 6 (2021): 688–718. 33287561 10.1177/1049909120966585 PMC8083078

[17] A. Brinkman‐Stoppelenburg , J. A. Rietjens , and A. van der Heide , “The Effects of Advance Care Planning on End‐Of‐Life Care: A Systematic Review,” Palliative Medicine 28, no. 8 (2014): 1000–1025. 24651708 10.1177/0269216314526272

[18] K. L. Harrison , E. R. Adrion , C. S. Ritchie , R. L. Sudore , and A. K. Smith , “Low Completion and Disparities in Advance Care Planning Activities Among Older Medicare Beneficiaries,” JAMA Internal Medicine 176, no. 12 (2016): 1872–1875. 27802496 10.1001/jamainternmed.2016.6751 PMC5304942

[19] D. Dobbs , N. S. Park , Y. Jang , and H. Meng , “Awareness and Completion of Advance Directives in Older Korean‐American Adults,” Journal of the American Geriatrics Society 63, no. 3 (2015): 565–570. 25803787 10.1111/jgs.13309 PMC4372806

[20] T. J. Bollyky , E. Castro , A. Y. Aravkin , et al., “Assessing COVID‐19 Pandemic Policies and Behaviours and Their Economic and Educational Trade‐Offs Across US States From Jan 1, 2020, to July 31, 2022: An Observational Analysis,” Lancet 401, no. 10385 (2023): 1341–1360. 36966780 10.1016/S0140-6736(23)00461-0 PMC10036128

[21] G. T. Anderson , “Let's Talk About ACP Pilot Study: A Culturally‐Responsive Approach to Advance Care Planning Education in African‐American Communities,” Journal of Social Work in End‐of‐Life & Palliative Care 17, no. 4 (2021): 267–277. 34605361 10.1080/15524256.2021.1976354

[22] J. J. Sanders , K. S. Johnson , K. Cannady , et al., “From Barriers to Assets: Rethinking Factors Impacting Advance Care Planning for African Americans,” Journal of Palliative Medicine 17, no. 3 (2019): 306–313.16. 10.1017/S147895151800038X 29869594

[23] T. P. Daaleman , C. P. Emmett , D. Dobbs , and S. W. Williams , “An Exploratory Study of Advance Care Planning in African‐American Elders,” Journal of the National Medical Association 100, no. 12 (2008): 1457–1462. 19110915 10.1016/s0027-9684(15)31547-9

[24] M. Hong , E. H. Yi , K. J. Johnson , and M. E. Adamek , “Facilitators and Barriers for Advance Care Planning Among Ethnic and Racial Minorities in the US: A Systematic Review of the Current Literature,” Journal of Immigrant and Minority Health 20 (2018): 1277–1287. 29124502 10.1007/s10903-017-0670-9

[25] R. L. Rhodes , N. J. Barrett , D. B. Ejem , et al., “A Review of Race and Ethnicity in Hospice and Palliative Medicine Research: Representation Matters,” Journal of Pain and Symptom Management 64, no. 5 (2022): e289–e299, 10.1016/j.jpainsymman.2022.07.009. 35905937

