# Supplementary material for: Disparities in Advance Care Planning: Did COVID‐19 Change Anything?
Source: J Am Geriatr Soc. 2026 May 13;74(6):1722–8. doi: 10.1111/jgs.70509 (PMC13266419; doi:10.1111/jgs.70509)
Supplement: Supplementary file 1 — Figure S1: Participant flow diagram. Table S1: Patient characteristics by race. Table S2: Frequency of talking to friends, family, or doctor about COVID‐19 related treatment preferences by willingness to talk, by race (n = 416). Table S3: Cross‐tabulation of preference for life‐prolonging treatment by discussions with friends, family, or doctors, stratified by race. Text S1: EQUAL‐ACP brief COVID‐19 survey. [file JGS-74-1722-s001.pdf]

Supplementary Table S1. Timing of COVID-19 Survey Completion by Race

COVID-19 Survey Timepoint	Black Older Adults (N=239)	White Older Adults (N=189)	Total (N=428)	p-value
Baseline	150 (62.8%)	127 (67.2%)	277 (64.7%)	
3 Months	27 (11.3%)	17 (9.0%)	44 (10.3%)	
6 Months	21 (8.8%)	14 (7.4%)	35 (8.2%)	
12 Months	41 (17.2%)	31 (16.4%)	72 (16.8%)	
**Overall Comparison**				**0.77**[1]

[1] P-value calculated using chi-square test.

COVID-19 survey responses were collected during scheduled data collection timepoints of the parent trial, the EQUAL ACP trial (Reducing Disparities in the Quality of Palliative Care for Older African Americans through Improved ACP), a multi-site cluster randomized trial of two advance care planning (ACP) interventions. Data were obtained at baseline (at or shortly after enrollment), and at 3, 6, and 12 months post-enrollment. The ACP intervention was delivered between baseline and the 3-month follow-up.

Supplementary text S1. EQUAL-ACP Brief COVID-19 Survey

1. How has the COVID-19 pandemic changed your willingness to talk to family, friends, or doctors about the medical care that you would want if you became too sick to speak for yourself? Compared to before the pandemic: are you (a) more willing; (b) less willing; or (c) no change in your willingness to talk about your wishes?
2. Have you talked to family or friends about the kind of medical care that you would want if you got COVID- 19? (a) Yes (b) No
3. Have you talked to your doctor about the kind of medical care that you would want if you got COVID- 19? Responses: (a) Yes (b) No
4. If you became very sick with COVID-19 and the doctors thought that you were not likely to recover, what kind of medical treatment would you want? (a) You would want all medical treatments to keep you alive as long as possible; (b) You would not want any medical treatments to prolong your life. Keep you comfortable and allow you to die naturally; (c) You would want the doctors to try medical treatments to keep you alive, but if you do not improve, stop life support treatments and keep you comfortable; allow you to die naturally.

Supplementary figure S1: Participant Flow Diagram

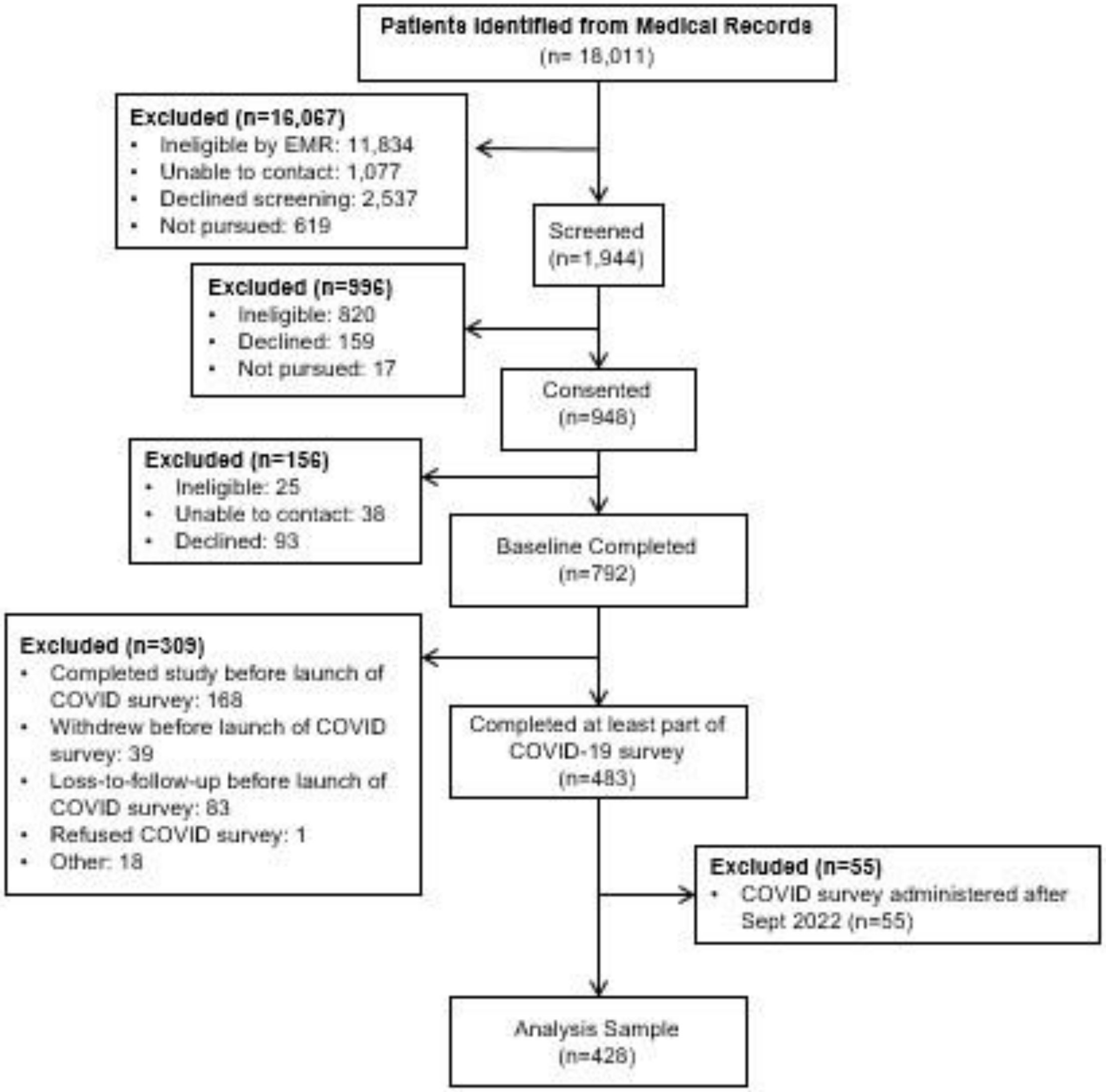

Supplementary table S2 Frequency of talking to friends, family, or doctor about COVID-19 related treatment preferences by change in willingness to talk, by race (n=416)

	Black Older Adults		*White Older Adults*		Overall	
	Less willing to talk or no change	*More willing to talk*	*Less willing to talk or no change*	*More willing to talk*	*Less willing to talk or no change*	*More willing to talk*
	n (%)	*n (%)*	*n (%)*	*n (%)*	*n (%)*	*n (%)*
Talked to friends, family, or doctor?						
No	142 (79%)	33 (65%)	119 (74%)	14 (54%)	261 (77%)	47 (61%)
Yes	37 (21%)	18 (35%)	41 (26%)	12 (46%)	78 (23%)	30 (39%)

As part of the COVID survey, participants were asked, “How has the COVID-19 pandemic changed your willingness to talk to family, friends, or doctors about the medical care that you would want if you became too sick to speak for yourself? Have you talked to family or friends about the kind of medical care that you would want if you got COVID- 19? (a) Yes (b) No

*Chi-square test on overall relationship between willingness to talk and frequency of talking (p=0.0039) *Breslow-Day Test for Homogeneity of the Odds Ratios by race p=0.876.

Supplementary Table S3. Treatment Preferences if Sick with COVID BY COVID-related ACP Discussions for each racial subgroup

	Black Older Adults (N=233)		White Older Adults (N=184)	
	Want life prolonging measures		Want life prolonging measures	
	Yes N (%)	No N (%)	Yes N (%)	No N (%)
**Talked to family, friends, or doctors**				
**Yes**	18 (27.3)	37 (22.2)	11 (57.9)	40 (24.2)
**No**	48 (72.7)	130 (77.8)	8 (42.1)	125 (75.8)

Want life prolonging measures (You would want all medical treatments to keep you alive as long as possible) versus other responses (You would not want any medical treatments to prolong your life. Keep you comfortable and allow you to die naturally; or You would want the doctors to try medical treatments to keep you alive, but if you do not improve, stop life support treatments and keep you comfortable; allow you to die naturally).

Talked to family, friends, or doctors: Combination of the following questions: Have you talked to family or friends about the kind of medical care that you would want if you got COVID-19? Have you talked to your doctor about the kind of medical care that you would want if you got COVID-19.
